# Single-cell epigenomics and proteomics methods integrated in multiomics

**DOI:** 10.1016/j.fmre.2023.11.014

**Published:** 2024-01-17

**Authors:** Haiyue Bi, Xiaocheng Weng

**Affiliations:** a Department of Clinical Laboratory, Center for Gene Diagnosis, and Program of Clinical Laboratory Medicine, Zhongnan Hospital of Wuhan University, Wuhan 430071, China; b College of Chemistry and Molecular Sciences, Key Laboratory of Biomedical Polymers of Ministry of Education Wuhan University, Wuhan 430072, China

**Keywords:** Single-cell multiomics, Multimodal omics, Epigenome, Cellular heterogeneity, Transcriptomics, Proteome

## Abstract

The meticulous examination of the genomic, transcriptomic, epigenomic, and proteomic landscapes, conducted at the precise resolution of single cells, has emerged as an indispensable instrument for comprehending the inherent mechanisms governing cellular heterogeneity. These methodologies have provided unprecedented insights into the intrinsic and extrinsic factors that underlie cellular morphological characteristics and differentiated functions. Within this field, multimodal techniques that concurrently analyze the epigenetic features of chromatin or cellular proteins and gene expression within an identical cell delineate intricate gene regulatory networks and phenotypes, thereby enhancing our understanding of cellular states during differentiation or pathological conditions. These techniques can be applied to identify cell subpopulations, infer cell developmental trajectories, and analyze patterns of cell-to-cell communication. In this context, we initiate by delineating the singular cell separation techniques employed in single-cell multiomics. Subsequently, we narrow our focus to methodologies amalgamating epigenetic features with gene expression at single-cell resolution. The epigenetic features entail DNA methylation, chromatin accessibility, histone modifications, chromatin conformation, and transcription factors. Following this, we discuss techniques for the conjoint analysis of cell surface and intracellular proteins in tandem with the transcriptome. Finally, we discuss the challenges and opportunities that manifest within this field, contributing to its continued advancement and exploration.

## 1 Introduction

Biological processes within organisms are intricately reliant upon cellular signaling and interactions, where the gene regulatory network imparts distinct physiological functions upon each individual cell. Consequently, a systematic exploration of gene regulatory networks within these cells has considerable potential for unraveling the mechanisms underlying the formation of genotype-phenotype disparities. It is widely acknowledged that all cell types in an organism share an identical genome. However, despite their morphological indistinguishability, diverse cell types undertake varied biological functions, and even subtle differences in gene expression levels emerge in response to external stimuli, giving rise to cellular heterogeneity [1]. Conventional methodologies employed for profiling genomics, transcriptomics, and other omics disciplines typically operate at the level of entire cell populations, which can obscure the inherent heterogeneity among individual cells. In the past ten years, advancements in single-cell techniques and high throughput sequencing have facilitated the individual profiling of genome [2], [3], [4] and transcriptome [5], [6], [7] in a meticulous, cell-by-cell manner or through highly parallelized approaches. These significant developments empower researchers to meticulously dissect the intricate functions and interactions that govern cellular behaviors.

**Fig. 1:**
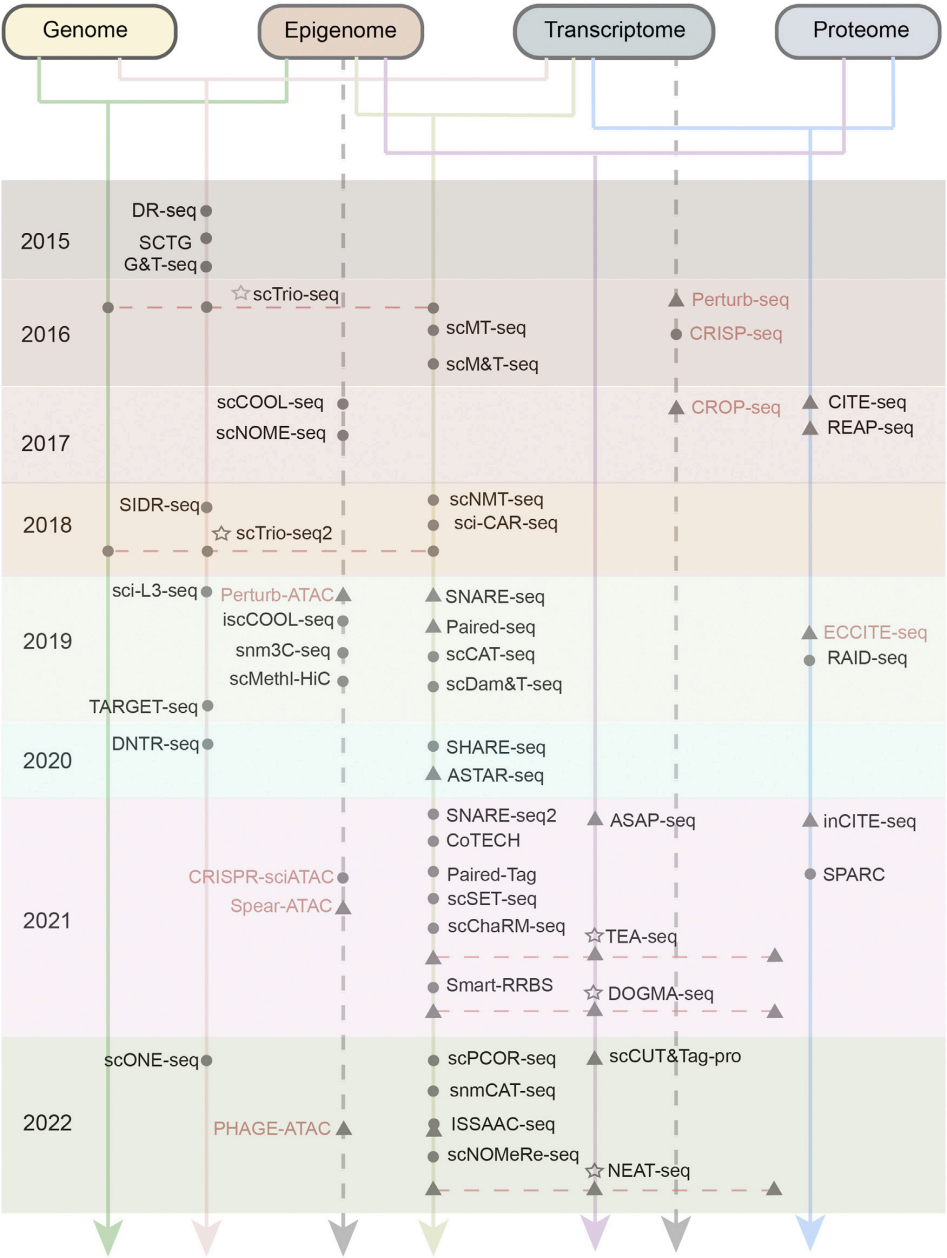
**Timeline of single-cell multimodal methods.** Here omics only includes genome, transcriptome, epigenome, and proteome, and methods only summarize the literature up to 2022. Methods appear on three vertical lines indicating that the profile three levels simultaneously. Gray dots represent plate-based methods; Gray triangles represent microfluidic assays. The five-pointed star represents the three-omics approach, and the levels involved are connected by dotted lines. Methods denoted with a red mark indicate the incorporation of expression perturbation within the respective techniques.

Intrinsically, cellular heterogeneity arises from a confluence of diverse factors, encompassed within various 'omics' layers, including but not limited to the genome, transcriptome, epigenome, and proteome. Recent advancements in single-cell multimodal omics and high-throughput sequencing platforms have facilitated the simultaneous profiling of two or more modalities (Fig. 1), rectifying the limitations of previous methodologies and providing an expansive and scalable toolkit for deconstructing the molecular circuits governing the functionality of distinct tissues and organs at the resolution of individual cells.

As the earliest single-cell multiomics technology, joint profiles of the genome and transcriptome can establish connections among single-nucleotide variants (SNVs), copy-number variants (CNVs), and gene expression, thereby elucidating the interplay between genetic mutations and cellular heterogeneity. These methods include G&T-seq (genome and transcriptome sequencing) [8], SIDR-seq (simultaneous isolation of genomic DNA and total RNA) [9], DNTR-seq (direct nuclear tagmentation and RNA sequencing) [10], DR-seq(gDNA-mRNA sequencing) [11], sci-L3-RNA/DNA (combinatorial indexing and linear amplification) [12], and scONE-seq [13]. However, these methodologies permit only the dissection of the transcriptional state of the cellular genome and do not afford insights into variations in cellular fate, meaning that they cannot elucidate why identical DNA sequences manifest divergent phenotypes across distinct cells. The remarkable phenotypic diversity among cells primarily stems from the epigenetic modifications that occur during gene expression. Excitedly, among the many existing single-cell multiomics techniques, progress has been made in experimentally elucidating and integrating epigenomic and transcriptomic data. This achievement provides a detailed and holistic representation that aids in deciphering the underlying regulatory principles governing cellular fate. Furthermore, transcriptomic analysis is exclusively focused on transcribed genes and is incapable of elucidating information regarding the abundance of proteins, which serve as the direct modulators of cellular functionalities. The current methods that integrate protein analysis into single-cell multiomics provide a more intuitive perspective for decoding cell lineage.

This review begins by presenting an overview of the prevailing techniques for single-cell isolation employed in single-cell multimodal-omics methodologies. Both low-throughput and high-throughput methodologies are discussed, as they constitute foundational yet pivotal steps in subsequent operations and data integration. Recent publications have provided an exhaustive overview of single-cell multiomics [14,15]. This review highlights the integration of experimental protocols and data from epigenomics and proteomics into single-cell multiomics approaches and further explores how these techniques shed light on the intricacies of cellular heterogeneity. Finally, it explores potential areas for improvement and extension within these methodologies, accompanied by suggestion of future prospects for the field.

## 2 Single cell isolation assays

**Fig. 2:**
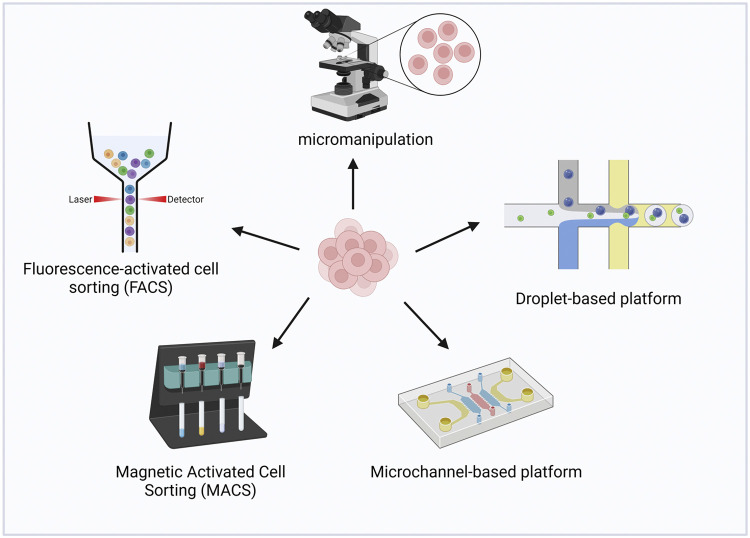
**Cell isolation methods adopted in single-cell multimodal methods.** Micromanipulation, fluorescence-activated cell sorting (FACS), and magnetic-activated cell sorting (MACS) are plate-based assays.

The isolation of individual cells is a prerequisite condition for the successful implementation of single-cell multiomics methodologies. It is imperative to employ separation methods that are both gentle and efficient to preserve the viability of cells for subsequent manipulations. Various factors, including cell type, cell quantity, and the design of the experimental protocol, dictate the selection of an appropriate cell isolation approach. Within the realm of single-cell multiomics strategies, the acquisition of single cells primarily relies on the utilization of the following two kinds of assays described below (Fig. 2).

### 2.1 Low throughput assays

Micromanipulation allows for the selection of cells with good morphology through microscopic observation and the aspiration of individual cells by pipetting technique. Complete morphology and almost undamaged cells can be obtained due to visualization operations [16]. This technique is handy and fast, but due to the specialized skills needed, only a few cells can be processed at a time, which is not conducive to high throughput methods. This approach is mainly used in the early stages of the development of single-cell multiomics. Techniques such as G&T-seq [8], DR-seq [11], scMT-seq (DNA methylome and transcriptome) [17], scCOOL-seq (Chromatin Overall Omic-scale Landscape Sequencing) [18], scChaRM-seq [19], and scNOMeRe-seq [20] are notable examples, which analyze one cell at a time.

Fluorescence-activated cell sorting (FACS) is a widely employed tool for the isolation of single cells within single-cell multiomics methodologies. This technique is used in methodologies such as scNOMe-seq (Nucleosome Occupancy and Methylome Sequencing) [21], snNMT-seq (single-cell nucleosome, methylation and transcription sequencing) [22], scDam&T-seq [23], Smart-RRBS [24], scONE-seq [13], and scPCOR-seq [25]. In these methodologies, cells undergo sorting and isolation into multiwell plates, ensuring the presence of only one cell per well prior to proceeding with subsequent steps. Target cells are labeled with fluorescent tags, which are subsequently recognized by fluorophore-coupled monoclonal antibodies. As a preprepared cell suspension flows through the cytometer, the cells are exposed to a laser, allowing the fluorescent detector to identify the fluorescent markers present on the cells [26]. Although flow cytometry is a commonly employed technique, it exhibits certain limitations. For instance, the requirement for a large number of input cells poses challenges when working with minute-scale samples, and the rapid flow within the machine can compromise the viability of sorted cells. Furthermore, the throughput may not be sufficiently high for handling large-scale samples.

An alternative approach known as magnetic activated cell sorting (MACS) relies on the binding of antibody-tethered magnetic beads to specific proteins present on target cells. By subjecting the cell suspension to a magnetic field, the magnetic beads attract the target cells, allowing for their easy isolation, while the unattracted cells are subsequently washed away [27]. However, it is important to note that this method is contingent upon the availability of suitable antibodies and is not universally applicable to all cell types, which restricts its broad utilization. In the context of scTrio-seq [28], MACS is integrated with micromanipulation techniques for sorting. Initially, a single-cell suspension is obtained using micromanipulation, after which MACS is employed to purify the cells, combining the advantages of both methodologies.

### 2.2 High throughput assays

While low-throughput single-cell multiomics analysis can offer high-quality data, the examination of highly heterogeneous tissues, such as brain tissue, necessitates methods capable of analyzing hundreds to millions of cells in parallel per experiment. In the context of single-cell multiomics analysis on these tissues, achieving substantial cell throughput becomes a pivotal requirement for accurate analysis. The cells derived from the tissue can be reliably identified only at a specific scale, which holds equal importance for both normal and pathological tissues [29]. A predominant obstacle in single-cell experimentation is the inherent data sparsity, a challenge that is particularly pronounced in single-cell epigenomics. While a cell houses hundreds of transcripts, facilitating transcriptome analysis, it contains merely two copies of DNA. The epigenetic signature must be discerned amidst the multitude of regulatory elements within these DNA copies. Consequently, there is an imperative to enhance both the sensitivity and throughput of single-cell methodologies. In recent years, the advent of microfluidic platforms utilizing various technologies has brought about a revolution in conventional approaches to single-cell acquisition [30]. These platforms have gained widespread adoption due to their inherent attributes of high throughput and robustness, particularly when dealing with bulk samples.

The pioneering commercially available microfluidic platform was the Fluidigm C1 platform, which utilized integrated fluidic circuits (IFCs) based on microflow-controlled chip technology. This microchannel-based platform enabled the processing of approximately 96 cells simultaneously. However, due to its inherent limitation in throughput, the platform was only implemented in a limited number of single-cell multiomics methods, such as Perturb-ATAC [31] and ASTAR-seq (assay for single-cell transcriptome and accessibility regions) [32], among numerous other published approaches. A droplet-based platform, has gained increasing prominence in the field of single-cell multiomics, owing to its capacity for high throughput and automation. The functionality of this platform primarily relies on the generation of nanoliter-scale gel bead-in-emulsions (GEMs), wherein individual cells are encapsulated together with barcoded oligonucleotide-coated beads. Following cell lysis, the barcoded analytes from multiple classes (including DNA, RNA, and protein) can be captured and subjected to subsequent processing. In addition to the barcodes assigned to each analyte, cell-specific barcodes are also linked to the beads, facilitating the unique identification of individual cells [33]. Various methodologies, such as Perturb-seq [34], CITE-seq [35], REAP-seq, ECCITE-seq [36], SNARE-seq [37], ASAP-seq, DOGMA-seq, TEA-seq [38], have been developed for this type of platform. While the throughput of this method is notably high, capable of analyzing up to 80,000 cells per run, it is important to note that the resulting cDNA library construction is unavoidably biased toward the 3′ end. In the realm of single-cell multiomics methodologies, the quantification of proteins of interest (POIs) is predominantly facilitated by antibodies conjugated with oligonucleotides on droplet-based platforms. Consequently, the resultant DNA and antibody libraries can be size-differentiated without compromising the integrity of other associated analyte libraries.

## 3 Link between the epigenome and gene expression

Gene expression is characterized by spatiotemporal specificity and cell-type particularity, predominantly modulated by cis-regulatory elements (CREs). An exhaustive elucidation of each cis-regulatory element is pivotal in deciphering gene regulatory mechanisms pertinent to species ontogenesis, cellular differentiation, and environmental adaptability [39]. Initial annotations of cis-regulatory elements (CREs) were derived predominantly from genomic sequence data. However, sequence information alone is insufficient to elucidate the dynamic role of CREs in orchestrating cellular functionalities and phenotypes. The process of gene transcription encompasses multifaceted interactions among various elements, including transcription factors, enhancers, promoters, and chromatin remodeling entities. Notably, these interactions are modulated by epigenetic alterations, encompassing DNA methylation, chromatin accessibility, histone modifications, and chromatin conformation [40].

Techniques in single-cell epigenomics, which are tailored to scrutinize single epigenetic features in isolation, have been instrumental in delineating cell-specific gene regulatory programs. However, analyzing individual features may provide only fractional insight into the intricate interplay of regulatory components. An integrative approach that concurrently assesses multiple omics layers at the single-cell level could furnish a more holistic comprehension of the interrelation between disparate epigenetic marks and gene expression [41]. However, transitioning from single omics to multiomics presents inherent challenges, notably the distinct experimental conditions and processing methodologies needed for detecting various epigenetic markers and conducting RNA sequencing. Harmonizing these experimental conditions is a salient concern. Furthermore, the order in which different epigenetic markers or omics analytes are processed warrants careful consideration. Recent advancements in multiomics methodologies have effectively addressed these concerns, illuminating numerous intricacies of cellular gene regulation.

### 3.1 Single-cell DNA methylation and gene expression

DNA methylation is a critical hallmark in gene expression regulation at the transcriptome level [42]. It primarily occurs at CpG sites, which are regions in the DNA sequence where a cytosine base is followed by a guanine base, forming a cytosine-guanine (CpG) dinucleotide. Within these CpG dinucleotides, DNA methyltransferases selectively methylate the 5th carbon of cytosine, leading to the formation of 5-methylcytosine (5mC) [43], which possesses the capacity to either enhance or suppress the affinity of cis-regulatory elements (CREs) for transcription factors, in a cell type-specific context. This modulation by 5mC has implications for a diverse array of biological processes and associated pathological conditions [44].

Two main sequencing methods are employed for DNA methylation analysis: single-cell reduced representative bisulfite sequencing (scRRBS) [45] and single-cell whole-genome bisulfite sequencing (scWGBS) (Table 1) [46]. Using these single-cell methylome sequencing methods, a large dataset of neuronal classifications in the human and mouse frontal cortex has been generated. Unlike single-cell RNA sequencing, single-cell methylome data allow for the prediction of cell type-specific regulatory elements from 5mC signatures. Single-cell parallel analysis methods for both the methylome and transcriptome have been developed, and the combination of the two can dissect the relationship between gene expression and the modification level of 5mC. For example, the technique of simultaneous analysis of the methylome and transcriptome was applied to study the heterogeneity and clonal evolution of chronic lymphocytic leukemia. The combined information at both omics levels confirmed that genetic subclones map to distinct clades, as inferred from the epimutation information derived from single-cell methylation sequencing alone [47].

**Table 1:** **Summary of single-cell methylation methods in multiomics**.

Basis	PBAT	Methods	Other data types	Cell type	Ref
scWGBS	Yes	scM&T-seq	mRNA, Smart-seq2	mESCs^a^	[51]
	Yes	scNOMe-seq	chromatin accessibility, GpC methyltransferase	GM12878 and K562	[21]
	Yes	scCOOL-seq	chromatin accessibility, GpC methyltransferase; CNV	mouse early embryos and mESCs	[18]
	YES	scNMT-seq	mRNA, Smart-seq2; chromatin accessibility, GpC methyltransferase;	mESCs	[22]
	No, TAILS	iscCOOL-seq	mRNA, scRNA-seq; chromatin accessibility, GpC methyltransferase	mouse oocytes	[52]
	No, TAILS	scChaRM-seq	mRNA, scRNA-seq; chromatin accessibility, GpC methyltransferase	human oocyte	[19]
scRRBS	No	scMT-seq	mRNA, Smart-seq2	sensory neurons	[53]
	No	scTrio-seq	mRNA, scRNA-seq; CNV	HHC^b^ tissue cell	[28]
	No	Smart-RRBS	mRNA, Smart-seq2	mouse embryos	[24]

a Mouse embryo stem cells.

b Human hepatocellular carcinoma.

Specifically, in scRRBS (reduced representation bisulfite sequencing), restriction enzymes are utilized to target CpG sites, making it a cost-effective alternative to scWGBS [48]. In the case of scM&T-seq (single-cell genome-wide methylome and transcriptome sequencing), a similar physical isolation approach to that of G&T-seq is employed, and scWGBS(single-cell whole genome sequencing) is utilized to generate methylation data. In scMT-seq, cytosolic RNA is manually isolated and then subjected to a modified Smart-seq2 procedure, while genomic DNA from the same cell undergoes RRBS. Smart-RRBS follows the same principles of library preparation for DNA methylome and transcriptome analysis as scMT-seq [24]. The key distinction lies in the separation process, where scMT-seq manually separates cytoplasmic RNA and nuclear DNA, while Smart-RRBS utilizes oligo(dT)-RT primer-conjugated magnetic beads for separation. scTrio-seq [28], similar to scMT-seq, requires manual separation of the cytoplasm and nuclei. This method has lower throughput than Smart-RRBS. In addition, these two methods use the original scRRBS method, which is time-consuming to obtain an ideal sequencing library. However, scTrio-seq concurrently examines single-cell genomes, methylomes, and transcriptomes, elucidating the relationships between copy number variation/DNA methylation and gene expression in both cancer cells and human colorectal cancer. Bisulfite conversion is universally employed to profile DNA methylation due to its robust conversion efficiency [49]. However, it is important to note that this method has the potential for DNA degradation due to the stringent reaction conditions [50]. In addition, the mapping rate of scWGBS is lower than that of RRBS. Unbiased sampling of the methylome by scWGBS does not enrich CpG-containing reads and CpG-rich gene regulatory regions, so the read coverage of unique CpGs of scM&T-seq is approximately three times less than that of Smart-RRBS.

### 3.2 Single-cell chromatin accesibility and gene expression

Through the modulation of nucleosome positioning and architecture, nucleosome remodeling complexes exert pivotal influence over transcriptional processes, encompassing initiation, elongation, and repression. Their actions either potentiate or attenuate gene expression contingent upon specific cellular contexts. Chromatin regions characterized by sparse nucleosome distribution—accessible chromatin regions—facilitate the binding of transcriptional regulatory elements to promoters and enhancers, thereby orchestrating gene expression dynamics and guiding intricate interactions throughout cellular differentiation and developmental trajectories [54,55]. Currently, there are two main tools for conventional bulk chromatin accessibility profiling: DNase-Seq [56] and ATAC-seq (Assay for Transposase Accessible Chromatin with high-throughput sequencing) [57]. Within the context of single-cell assays, the experimental protocol for DNase-Seq manifests significant complexity. Conversely, ATAC-seq employs Tn5 transposases integrated with adapters, a methodology that substantially streamlines the library construction process. Consequently, ATAC-seq has garnered widespread adoption to map candidate cis-regulatory elements in the realm of single-cell chromatin accessibility analyses [58]. In contemporary research, methodologies derived from ATAC-seq have been advanced for use in platforms encompassing microwell-based, nanowell-plate-based, microfluidic, droplet-based, and combinatorial barcoding systems [40]. These innovations have catalyzed the integrative analysis of chromatin accessibility alongside other omics analytes. For example, joint analysis of chromatin accessibility and the transcriptome at single-cell resolution can reveal elements responsible for driving gene expression in a cell type-specific manner during the cell maturation trajectory.

Recently, various dual-omics methodologies have been developed, combining Tn5-based approaches with transcriptomic profiling, albeit with different levels of throughput and sensitivity (Table 2). In scCAT-seq [59], single cells undergo physical separation of cytoplasmic RNA from nucleus. However, this method can only process one cell at a time, limiting its throughput. Additionally, the isolation of small amounts of DNA and RNA before amplification results in low-abundance genes, thus providing a partial landscape of the whole-cell transcriptome-wide information. Another approach, ASATR-seq (Single-cell protein and RNA co-profiling) [32], based on the Fluidigm C1 microfluidic platform, improves throughput by two orders of magnitude compared to scCAT-seq. After isolating single cells, Tn5 transposase assembled with sequencing adaptors targets accessible DNA regions before mRNA RT. Attempts were made to reverse the transposition and RT steps, but they were unsuccessful, likely due to Tn5 digestion of the cDNA, as confirmed in subsequent publications [60,61]. The droplet-based method, SNARE-seq [37], addresses the need for high throughput and automation. In this method, chromatin open regions in each permeabilized nucleus are tagmented by Tn5 transposase and then encapsulated within a droplet. This enables the inclusion of accessible genomic loci and mRNA from the same single nucleus within the same droplet. To separate fragmented chromatin and mRNA, a splint oligonucleotide is designed, with one end complementary to the Tn5 adapter sequence and the other end containing a polyA tail that can be captured by poly T-coated beads with a shared barcode. Because DNA and RNA from the same cell carry the shared barcode, subsequent data analysis does not require probabilistic mapping of single-cell clusters.

**Table 2:** **An overview of methods for joint profiling of single-cell chromatin accessibility and gene expression**.

Methods	Technology	Batch size	Case study	Amplification strategy	Ref.
Sci-CAR-seq	Two rounds of combinatorial indexing	4825 cells	11,296 cells from whole mouse kidneys	Independent amplification of indexed DNA and mRNA	[70]
scCAT-seq	manually/liquid-handling robot	176 cells	110 clinically discarded human embryos	Independent amplification of DNA and mRNA	[59]
SHARE-seq	Three rounds of combinatorial indexing	∼2100 cells	34,774 mouse skin cells, 23,278 GM12878 cells	Independent amplification of indexed DNA and mRNA	[71]
SNARE-seq	Droplet-based	1047 cells	5081 cells from mouse neonatal cerebral cortex, 10,309 cells from adult mouse cerebral cortex	Independent amplification of DNA and mRNA	[37]
Paired-seq	Five rounds of combinatorial indexing	2053 nucleis	15,191 nuclei from adult mouse cerebral cortex	Preamplification of Barcoded DNA and mRNA before split	[66]
ASTAR-seq	Based on fluidigm C1 microfluidic chip	96 cells	192 E14 mESCs	Independent amplification of DNA and mRNA	[32]
ISSAAC-seq	FACS or 10x Genomics	not mentioned	10,378 adult mouse cerebral cortex	Independent amplification of DNA and mRNA	[69]

**Fig. 3:**
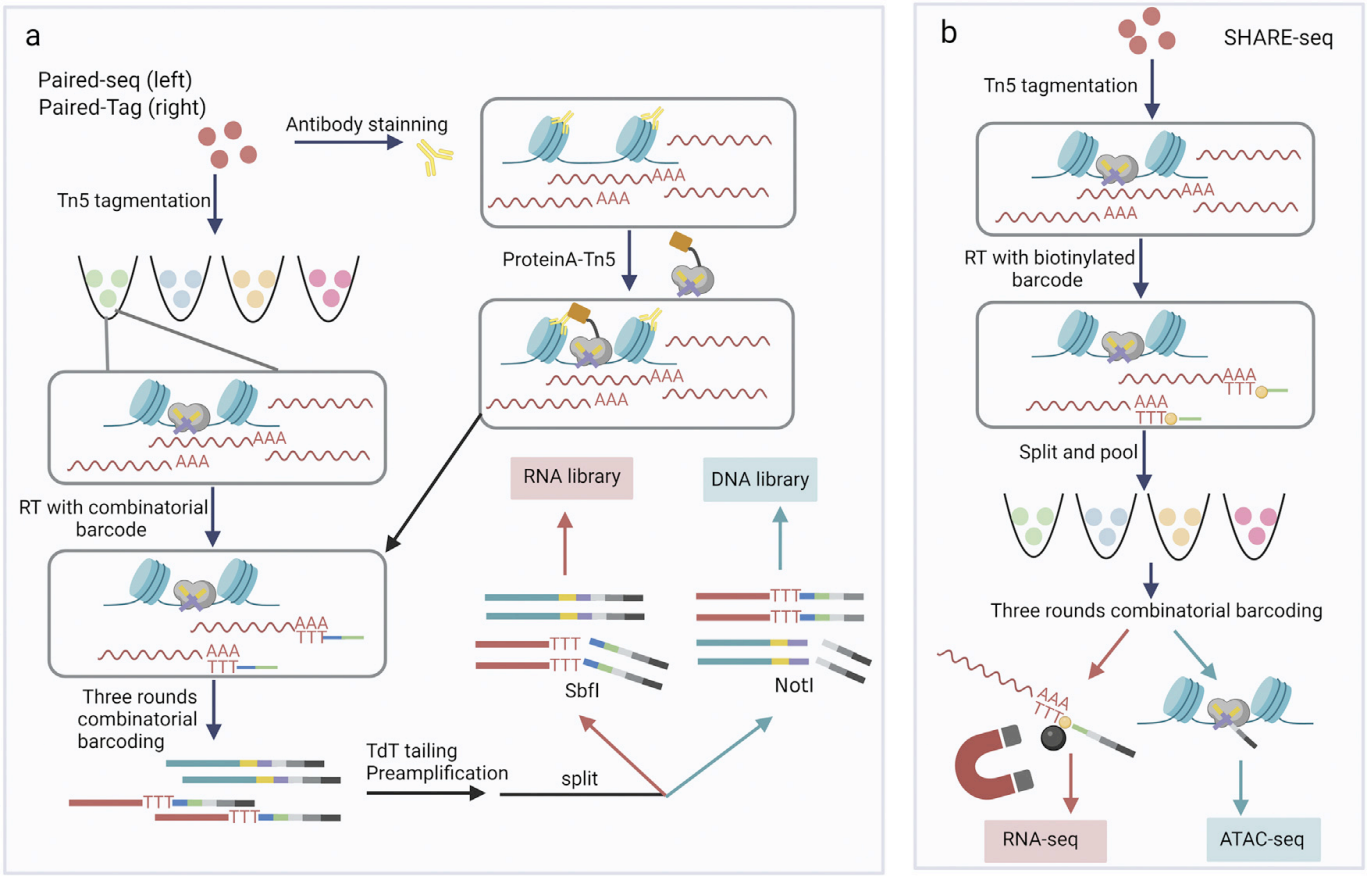
**Single-cell multiomics sequencing protocols for joint analyses of the epigenome and transcriptome in a combinatorial indexing manner.** (a) Paired-seq (left) [66] and Paired-tag (right) [78]. These two methods utilize combinatorial barcodes for reverse transcription. (b) SHARE-seq [71] utilizes a biotinylated barcode for reverse transcription. BC, barcode; TdT, terminal deoxynucleotidyl transferase; SbfI and NotI, restriction endonuclease.

As the research focus shifts toward the development of multi-omic single-cell atlases for heterogeneous organs and tissues, there is a growing demand for methods that offer higher cell throughput and produce error-free data of high quality. Single-cell combinatorial indexing ("sci") methods utilize split-pool barcoding to uniquely label the nucleic acid content of individual cells or nuclei [62], [63], [64], [65]. These methods provide a highly scalable alternative to droplet-based systems without requiring specific instrumentation. Several methods combining Tn5-based tagmentation of open chromatin with ``sci'' strategies have been developed. One notable method is sci-CAR(chromatin accessibility and mRNA), which represents a dividing line in the joint analysis of chromatin accessibility and the transcriptome, as previous methods relied on physically separating chromatin and mRNA. In sci-CAR, cells undergo the first round of reverse transcription (RT) and Tn5 indexing, after which they are pooled, lysed, and split for a second round of indexing. However, a critical drawback of this technology is that a single cell is divided into two aliquots to amplify DNA and RNA separately without preamplification, leading to a drop in capture efficiency. Additionally, the second round of indexing occurs after the split, which disrupts the cell structure. Consequently, only two rounds of indexing can be achieved at most, resulting in limited throughput. Paired-seq (parallel analysis of individual cells for RNA expression and DNA accessibility by sequencing) adopts a ligation-based combinatorial indexing strategy to tag accessible chromatin fragments generated by Tn5 and cDNA generated by RT in millions of cells [66]. The method introduces a “library dedicating” strategy (Fig. 3a), where different restriction endonucleases are used after splitting to obtain specific end sequences corresponding to amplification primers. This allows for the distinction between DNA and RNA, enabling the construction of dedicated libraries. Although the throughput of Paired-seq is improved, the complexity of the library is lower compared to stand-alone single-cell sequencing. Another technique, named SHARE-seq (simultaneous high-throughput ATAC and RNA expression with sequencing), is based on Paired-seq and SPLiT-seq [67]. It separates cDNA from whole DNA using streptavidin magnetic beads, enhancing capture efficiencies and library complexity. After three rounds of hybridization with well-specific barcoded oligonucleotides targeting transposed chromatin fragments and poly(T) cDNA (Fig. 3b), separate libraries are prepared for parallel sequencing. The advanced version of SNARE-seq, SNARE-seq2 [68], also adopts a ligation-based ``sci'' approach, replacing the droplet-based method. The simplicity of the sci-experimental setup offers scalability and flexibility for multisample processing.

Several high-throughput methods, such as sci-CAR, SNARE-seq2, Paired-seq, and SHARE-seq, have emerged that enable simultaneous processing of a large number of individual cells using combinatorial indexing. However, a challenge inherent to combinatorial indexing is the unavoidable collision rate, which hinders the precise indexing of each cell for achieving a downstream one-to-one correspondence between open chromatin DNA and mRNA-generated DNA within the same single cell. Consequently, some cells may end up with the same indexing, leading to ambiguity in the subsequent analysis. Although the collision rate can be reduced by increasing the complexity of the index and reducing the number of cells per well during the splitting step, such approaches come at the expense of higher experimental costs. Moreover, these high-throughput methods often generate datasets that exhibit a scarcity of sequencing reads for each profiled cell, resulting in significant information loss. Therefore, if the research objective is to delve deeper into the molecular distinctions between cell subpopulations, analyzing one cell at a time may be more favorable. This single-cell analysis approach allows for a more comprehensive and accurate assessment of the molecular characteristics within each individual cell, ensuring a more thorough exploration of the entire cellular landscape.

A more recent method called ISSAAC-seq (in situ sequencing hetero RNA-DNA hybrid after assay for transposase-accessible chromatin-sequencing), built upon the SHERRY (Sequencing HEteRo RNA-DNA-hYbrid) and scATAC-seq technique [60,61], has been developed, which is compatible with both low and high-throughput library construction platforms. [69]. ISSAAC-seq encompasses three distinct modules. The last two modules are the cell isolation module and the library construction module, which can be tailored according to the specific research objectives and characteristics of the samples. For cell isolation, either flow cytometry, suitable for a limited number of cells, or microfluidics-based techniques, suitable for high-throughput studies, can be employed in conjunction with ISSAAC-seq. This method exhibits promising potential in single-cell multiomics sequencing, as it can be combined with other approaches to analyze multiple omics levels, extending beyond chromatin accessibility and transcriptome analysis. Overall, ISSAAC-seq presents a valuable avenue for comprehensive and integrated single-cell multiomics sequencing, with the flexibility to investigate various omics layers and accommodate diverse research needs and sample types.

### 3.3 Single-cell histone modifications/transcription factor (TF) binding and gene expression

The dynamics of the chromatin landscape exert a profound influence on gene expression. Perturbations in the epigenetic processes can precipitate alterations in histone modifications and DNA, either enhancing or repressing specific gene expression profiles, which may cause various diseases. During the course of mammalian cellular development, concomitant histone modifications—those indicative of gene activation (H3K4me3) and those associated with gene repression (H3K27me3)—are discernible in the promoter regions of developmental genes. This bivalent chromatin modification state is thought to provide important regulatory genes for expression or repression during cell lineage specification [72]. ChIP-seq is widely adapted to profile histone modifications in bulk cells in the earlier stage. However, processing single cells using ChIP-seq is challenging because chromatin immunoprecipitation is very inefficient. Subsequently the developed CUT&RUN (pA/G-MNase) [73] and CUT&Tag (pA-Tn5) [74] technologies used enzymes that could target sites of interest in situ to fragment chromatin, improving efficiency and the signal-to-noise ratio. However, to avoid the innate binding of pA-Tn5 to open regions of chromatin, high-salt conditions were used in the CUT&Tag protocol, which makes it unsuitable for the analysis of transcription factors. In protocols adapted to single cells, ultralow input CUT&RUN (uliCUT&RUN) identifies TF binding sites in developing embryos [75]. scCUT&Tag and droplet-based 10x scCUT&Tag have been employed in both mouse brain tissue and human brain tumors to delineate histone modifications. These endeavors facilitate the elucidation of cellular identity and underpin the decoding of regulatory principles [76,77].

Based on the above single-cell technologies, several multiomics technologies that simultaneously analyze histone modifications and gene expression have been developed. Paired-tag [78] (Fig. 3a) and coTECH (combined assay of transcriptome and enriched chromatin binding) [79], employing split-pool strategies, allow for the joint profiling of histone modifications and the transcriptome in a high-throughput manner. In the scSET-seq methodology [80], an initial separation of the nucleus and cytoplasm is executed. Following this separation, pA-Tn5 is employed for the subsequent stages of library construction. While this procedure can result in quantifiable sample attrition, it significantly mitigates the potential for cross-contamination between the DNA and cDNA libraries. These methodologies provide invaluable insights into the modulation of gene expression governed by epigenetic characteristics. Specifically, Paired-tag is used to profile five histone modifications jointly with the transcriptome in the adult mouse frontal cortex and hippocampus and identify distinct epigenetic regulatory processes acting at different categories of genes and CREs. CoTECH disentangles the context-specific interplay between H3K4me3/H3K27me3 and transcription level.

### 3.4 Profiling multiple epigenome features

#### 3.4.1 Joint profiling of DNA methylation and chromatin accessibility

Distinct DNA methylation profiles are observed across various cell types [81,82], indicative of its dynamic regulatory nature. Investigating the interplay between DNA methylation and chromatin accessibility, particularly in primordial germ cells or embryonic stem cells, is pivotal for elucidating the mechanisms underlying development and differentiation. The simultaneous profiling of DNA methylation and chromatin accessibility at the single-cell level is made possible by scCOOL-seq [18], a method that combines scNOMe-seq [21] and PBAT-seq (Post-Bisulfite Adaptor Tagging Sequencing) [50]. This approach, known as iscCOOL-seq [52], eliminates tailing and ligation steps for individual cells, reducing the cost of single-cell DNA methylome library preparation and improving mapping efficiencies. This method dissects the complex, yet highly coordinated, epigenetic alterations during mouse oocyte growth and the establishment of totipotency, which remain inscrutable when relying solely on individual epigenetic markers. Another method developed by the same research group, called scChaRM-seq, combines iscCOOL-seq with scRNA-seq to enable the simultaneous profiling of the DNA methylome, chromatin accessibility, and transcriptome [19]. In addition, scNMT-seq is capable of the simultaneous identification of these three levels at single-cell resolution and dissects the dynamics of epigenome interactions during a developmental trajectory in mouse embryonic stem cells [22]. Among the mentioned methods, scChaRM-seq and scNMT-seq analyze chromatin accessibility and the transcriptome simultaneously, utilizing bisulfite conversion to encode CpG accessibility and distinguish between inaccessible chromatin and unavailable data. It is important to note that the resolution of these methods depends on the frequency of CpG sites in genomic DNA rather than the size of the library fragments. However, both scChaRM-seq and scNMT-seq rely on BS-seq and inherit its limitations, including potential DNA damage, which can affect sequencing accuracy and sensitivity. Consequently, the use of methyltransferase-based approaches is not suitable for profiling chromatin accessibility and occupancy.

#### 3.4.2 Joint profiling of DNA methylation and chromatin conformation

Single-cell chromatin conformation analysis techniques enable the investigation of a plethora of issues pertaining to 3D genomes at the individual cell level, encompassing interactions between enhancers and promoters as well as intricate three-dimensional genomic structural attributes such as compartments, TADs (Topologically associating Domains), and loops [83]. Single-cell chromatin conformation capture (3C) and Hi-C are emblematic methodologies [84,85]. However, while these techniques permit profiling of cell cycle patterns, they have not yet been refined to achieve cell type-specific investigations. Methyl-HiC and sn-m3C-seq integrate DNA methylation sequencing, capable of discerning cell subpopulations, with chromatin conformation analysis. This integrative approach enables the detailed elucidation of cell type-specific chromatin structural dynamics within heterogeneous cells and can aid in the reconstruction of chromatin conformational landscapes for specific cellular phenotypes. A recent study combined snmC-seq3 and sn-m3C-seq to establish the first brain-wide, single-cell resolution DNA methylome and 3D multiomic atlas, which offers an unrivaled repository for understanding the cellular-spatial and regulatory genomic diversity of the mouse brain [86].

The aforementioned analyses predominantly focus on the chromatin level. However, the delineation of cell identity necessitates an integrative examination across gene expression and diverse omic layers. Contemporary investigations into chromatin conformation have yet to incorporate gene expression into their purview. The novel method termed HiRES [87], however, synergizes chromatin conformational analysis with gene expression, revealing significant chromatin reconfiguration preceding transcriptional activation. This underscores the intricate interplay between chromatin interactions and transcriptional regulation.

#### 3.4.3 Multiplexed profiling of histone modifications

In prior methodologies, concurrent analyses were conducted on the associations between histone modifications or transcription factors and the transcriptome. However, they did not elucidate the mechanisms of how multiple regulatory proteins collaboratively operate on CREs to determine transcriptional outcomes. Consequently, the concomitant interplay among diverse chromatin attributes, specifically between various histone modifications and transcription factors, holds equivalent significance. To map multiple chromatin features in the same cells at the same time, in advanced methodologies such as Multi-CUT&Tag [88] and MulTI-Tag [89], barcoded antibodies are strategically integrated with pA-Tn5 to concurrently target distinct histone modifications or chromatin-associated proteins. Compared with CUT&Tag, these two methods have comparable sensitivity and signal-to-noise ratios. In addition, both the Multi-CUT&Tag and MulTI-Tag methodologies elucidate the shared associations between RNAPII and diverse histone modifications. Additionally, they shed light on the interplay between active and repressed histone modifications, proving instrumental in analyzing distinct cell types and delineating differentiation pathways. The CUT&Tag2for1 methodology, through the integration of a mixture of pertinent antibodies, is adept at simultaneously profiling RNA polymerase II and H3K27me3. This approach yields high-resolution mappings of both active and repressive regulomes within individual cells [90].

### 3.5 CRISPR-based single-cell dual-modal omics

The CRISPR system is an efficient genome editing toolkit, that has further facilitated gene function studies and immune-oncology (IO) target discovery. By integrating CRISPR perturbations with gene expression or chromatin accessibility profiling, it becomes possible to simultaneously investigate the function of multiple genetic elements and explore their interplay. Pioneering CRISPR-based methods such as CRISP-seq [91] and Perturb-seq [34] enable high-throughput transcriptome profiling by incorporating a guide barcode into a plasmid encoding single-guide RNA (sgRNA). Subsequently, mRNA and barcoded sgRNA are subjected to reverse transcription (RT) along with a cell barcode, utilizing droplet-based platforms.

While these methods have demonstrated effectiveness, there is a potential risk of mismatches between the guide RNA (gRNA) and barcode as the number of gRNA libraries increases. Recent advancements, such as CROP-seq [92], have addressed this concern by directly sequencing gRNA in a manner similar to single-cell RNA sequencing, utilizing a regular poly-T approach. Other scCRIPR methods integrated with ATAC have also made significant progress in studying chromatin accessibility changes in perturbed cells, opening up a new avenue for dissecting mechanisms of epigenetic regulation. Examples of such methods include Perturb-ATAC [31], CRISPR-sciATAC [93], and Spear-ATAC [94] (Table 3).

**Table 3:** **Overview of CRISPR-based methods in single-cell dual-modal omics**.

Assay	sgRNA identification	perturbation mechanism	cell numbers	omics	Ref.
Perturb-seq	Barcode	CRISPRi^a^	15 006	Transcriptome	[34]
CRISP-seq	Barcode	Cas9ko^b^	6144	Transcriptome	[91]
CROP-seq	Polyadenylated sgRNA	Cas9ko	5798	Transcriptome	[92]
CRISPR-sciATAC	Polyadenylated sgRNA	Cas9ko	16 676	Chromatin accessibility	[93]
Spear-ATAC	sgRNA in genome	CRISPRi, Cas9ko	32 832	Chromatin accessibility	[94]
Perturb-ATAC-seq	Barcode	CRISPRi, Cas9ko	2627	Chromatin accessibility	[31]
ECCITE-seq	sgRNA	Cas9ko	4120	proteome, transcriptome	[36]

a CRISPR-based genetic interference.

b Cas9-based knockout.

## 4 Link between single-cell protein measurement and gene expression

According to the foundational principles of the central dogma, gene expression encompasses the transcription of DNA into mRNA, followed by the translation of mRNA into proteins. Serving as a pivotal intermediary within this paradigm, RNA holds an essential position within gene regulatory networks. Concurrently, proteins, executing cellular functionalities, are indispensable molecular entities within cellular systems. In recent advancements, single-cell transcriptomic methodologies have emerged as pivotal tools in elucidating intercellular heterogeneity and delving into the intricacies of cellular dynamics within multifaceted biological systems [95,96]. However, transcripts and proteins are not necessarily related due to changes in gene expression regulation [97]. To achieve a deeper understanding of cell type-specific phenotypes and functionalities, it is imperative to leverage single-cell protein analysis. These sophisticated analytical techniques can elucidate intricate variations associated with cell fate determination, signal transduction, and disease development. Concomitant examination of single-cell proteins and transcripts enables an in-depth exploration of the interplay between gene expression heterogeneity and phenotypic variation, potentially revealing previously unidentified cell subpopulations and elucidating their functional roles. In addition, in a number of contemporary multiomics methodologies, spanning the central dogma has been achieved by combining gene expression, chromatin accessibility, and quantitation of intracellular or cell surface proteins.

At present, there are two predominant methodologies for protein quantification in single-cell multiomics: one rooted in single-cell mass spectrometry (scMS) and the other leveraging next-generation sequencing (NGS) technology [98]. The latter is widely used in single-cell multiomics technology. Primarily, in methods based on next-generation sequencing (NGS), each antibody specific to an epitope is conjugated to an oligonucleotide. This permits its conversion into a DNA sequence suitable for library preparation and subsequent NGS alongside other analytes. Consequently, this process transforms nonamplifiable protein quantification data into amplifiable library information, harmonizing it with existing genomic and transcriptomic sequencing platforms. The application of single-cell mass spectrometry has been constrained to a narrow range of cell types due to its inability to amplify proteins and its specific requirements for sample preparation and instrumentation. While single-cell genome and transcriptome sequencing have experienced extensive advancement in multiomics modalities, facilitated by high-throughput single-cell platforms, the adoption of scMS remains limited. This is predominantly attributed to the constraints of the existing scMS experimental protocols, which have limited throughput for the number of cells that can be analyzed. However, recent technological advancements in single-cell mass spectrometry facilitate the analysis of multiple proteins within an individual cell, obviating the need for affinity reagents, and thereby enhancing its appeal for single-cell multiomics methodologies [99], [100], [101]. In the following section, we will discuss single-cell multiomics methodologies that incorporate protein measurement. These can be principally categorized into techniques based on scMS and those based on NGS methodologies.

### 4.1 Oligonucleotide barcoded antibody-assisted protein measurement

The distinct biochemical properties of nucleic acids and proteins pose challenges for protein sequencing at single-cell resolution, thus hindering its development. Traditionally, protein measurements have relied on antibody-based detection or mass spectrometry. However, a clever solution for protein quantification has emerged through the conjugation of sequencing oligonucleotides with antibodies specific to target proteins. Barcoded antibody-based methods enable the generation of dozens to hundreds of encoded antibodies capable of recognizing epitopes or proteins of interest in thousands of individual cells. These antibodies are associated with barcodes that can be sequenced along with endogenous mRNA, allowing for subsequent protein quantification by counting the antibody barcodes. Most of these methods are performed on droplet-based platforms, which streamline manual operations. Barcoded antibody-based methods can be bifurcated based on the targeted protein: one focuses on the quantification of cell surface proteins, while the other centers on the quantification of intracellular proteins.

#### 4.1.1 Detection of cell surface proteins

**Fig. 4:**
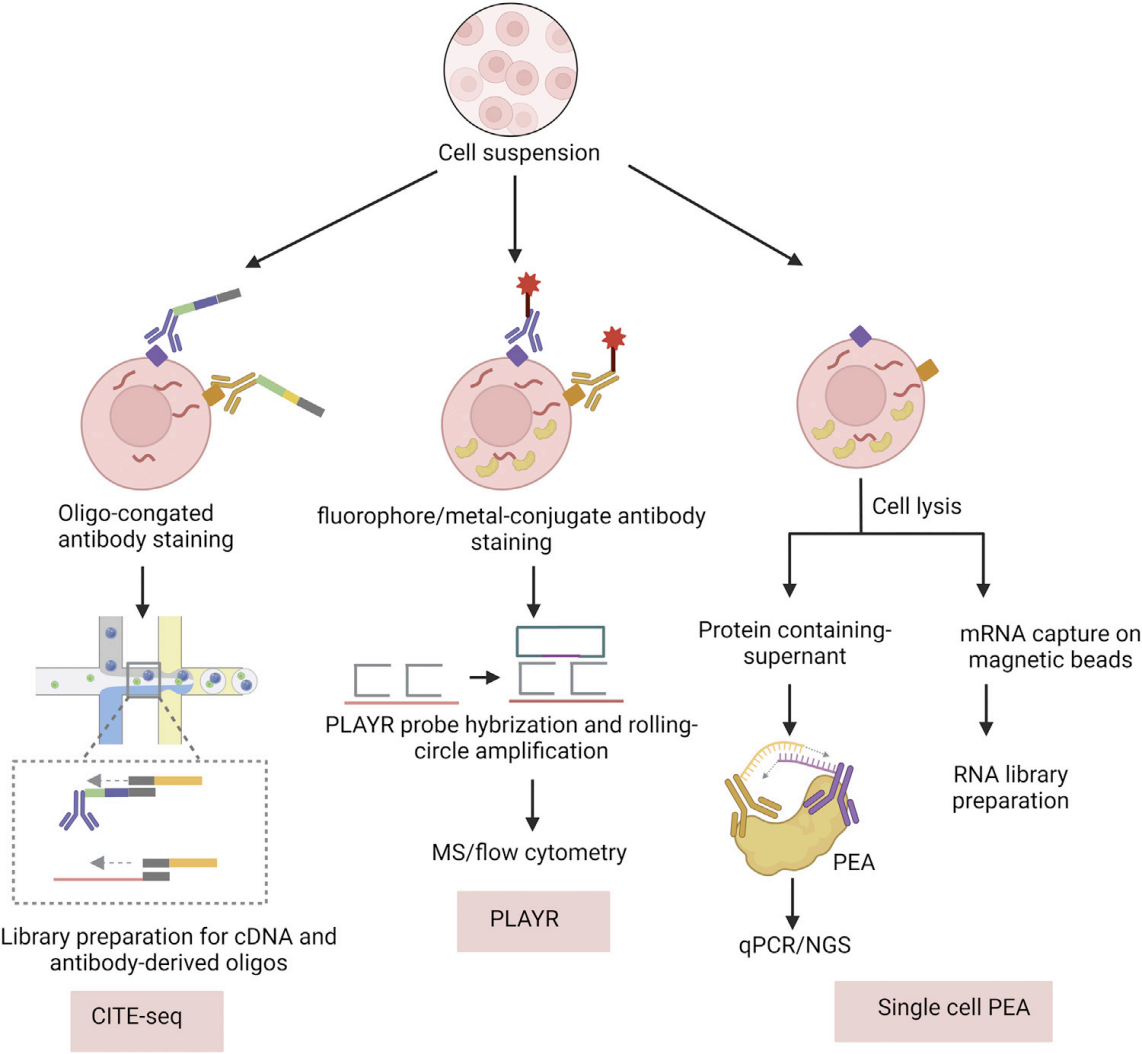
**Single-cell multi-omics sequencing protocols for joint profiling of protein and transcriptome.** CITE-seq [35] targets single-cell surface proteins by oligonucleotide-conjugated antibodies, and then the antibody-derived tag and mRNA are captured separately on the droplet-based platform. PLAYR [143] labels cell surface proteins with antibodies conjugated with fluorophores or metals, while RNA is stained by PLAYR probes. PEA-based methods, such as SPARC [111] and RAID-seq [108], use a mixture of protein-specific PEA probes that can be quantified using qPCR or NGS. MS, mass spectrometry. PEA, proximity extension assays.

Flow cytometry remains the benchmark technique for delineating cell subpopulations through quantitative variations in surface markers [102]. However, its compatibility with several contemporary high-throughput single-cell platforms is limited. The advent of CITE-seq (cellular indexing of transcriptomes and epitopes by sequencing) effectively addresses this limitation. This methodology has subsequently been commercialized and has gained prominence in single-cell multiomics research [35]. The antibody utilized in CITE-seq is conjugated to well-designed oligonucleotides using a disulfide bond that can be broken under reduced conditions. The conjugate includes a barcode for antibody identification, a handle for PCR amplification, and poly(A) tails similar to mRNA, enabling their capture by oligo-dT primers. In CITE-seq, cells recognized by the antibody and drop-seq beads are encapsulated together. After cell lysis, oligo-dT-bearing beads with shared cell identifiers capture both antibodies and mRNA during reverse transcription. The resulting amplified cDNAs and antibody-derived tags (ADTs) can then be converted into next-generation sequencing (NGS) libraries (Fig. 4). Similar approaches are employed in REAP-seq [103] and ASAP-seq [104], with slight variations in barcode structure and ligation manner. When integrated with scRNA-seq, CITE-seq has the capability to discern immune cell phenotypes that remain elusive to scRNA-seq analysis alone. Through the employment of CITE-seq for immunophenotypic analysis, scholars have delineated novel PD-L1/PD-L2+ macrophage subsets within human breast cancer cells that have a significant correlation with clinical prognosis [105]. In addition to joint analysis with gene expression, identification of cell surface proteins and combined analysis of protein-DNA interactions can provide insights into the relationship between immune cell states and genomic regulatory elements, further identifying extensive and cell-type-specific regulatory priming [106].

Despite the advancements in commercial CITE-seq panels, which have enhanced signal detection and reduced background interference through meticulous optimization of antibody concentration and volume [107], certain challenges persist. Given that the sequencing signal might originate from residual antibody conjugates, multiple washes of the cells are imperative. However, this frequent washing can diminish the cell count, necessitating an increase in the initial cell number, which, in turn, might elevate the background noise. Striking an optimal balance between cell count and washing frequency presents a considerable challenge. Furthermore, the applicability of CITE-seq is circumscribed by the extant antibodies and labeling methodologies. The repertoire of cellular epitopes amenable to labeling remains finite. However, the most significant drawback of CITE-seq and similar methods lies in antibody cross-reactivity, which is particularly exacerbated in the context of higher multiplexed profiles.

#### 4.1.2 Detection of intracellular proteins

CITE-seq and similar methods are predominantly suitable for immune cells. However, they are not applicable to nonimmune cells and tissues requiring dissociation, as these techniques compromise the integrity of the cell membrane. Immunostaining of intracellular proteins necessitates cell fixation and permeabilization, which can potentially cause invasive damage. In RAID-seq (single-cell RNA and Immuno-detection) [108], reversible fixation is performed, allowing for intracellular immunostaining with RNA-barcoded antibodies and joint profiling with the transcriptome. Aside from reversible fixation, a PEA ( proximity extension assay) combined with Smart-seq2 presents a noteworthy method for simultaneously measuring mRNA and intracellular proteins in a single cell (Fig. 4). PEA is a homogeneous affinity-based method optimized from the PLA (proximity ligation assay) that requires a pair of adjacent oligonucleotide-labeled antibodies to generate a DNA reporter for protein detection. Compared with protein detection methods based on single antibodies such as CITE-seq, this method has reduced background and nonspecific adsorption, which is an ideal solution to reduce cross-reactivity [109,110]. However, RAID-seq is limited to processing only six proteins at a time. Another method, SPARC (Single-Cell Protein And RNA Co-profiling), which omits cell fixation, integrates the multiplex PEA panel, enabling precise transcript quantification and scalable analysis of 89 intracellular proteins. However, it remains challenging to elucidate the connection between nuclear proteins and RNA in immune-free cells or cells with damaged membranes, as the adhesion environment within the nucleus can lead to nonspecific antibody binding [111]. In inCITE-seq, carried out on a droplet-based profiling platform, optimized blocking buffers minimize nonspecific bindings, achieving high throughput and specificity for individual nuclei profiles [112]. Furthermore, the incorporation of nucleus-hashing antibodies in this methodology allows for multiplexing analysis of multiple samples [113]. Another noteworthy and unconventional method is PHAGE-ATAC, a droplet-based single-cell ATAC-sequencing and protein quantification method. Phage-encoded nanobodies, which possess the ability to recognize antigens on the cell surface and index ATAC fragments, play a key role in this method. The unique genetic barcode contained within each phage particle's CDR3 (hypervariable complementarity-determining region 3) enables the mapping of protein quantities and open chromatin information for each cell using the resulting PDT (phage-derived tag) library and ATAC-seq library.

These methodologies furnish unparalleled insights into the relationships between intracellular protein and gene expression, insights that are unattainable through techniques predicated on cell surface protein analysis. Specifically, RAID-seq successfully recapitulated differentiation-state changes at the protein and mRNA levels in human keratinocytes. The epitopes identified by RAID-seq also include two phosphorylated epitopes, which are related to signal transduction processes related to cell differentiation. Despite these initial approaches, several challenges remain in combining intracellular multiplex protein determination and nucleic acid analysis. The specificity of antibodies and nanobodies limits the accuracy and depth of data generated by the aforementioned methods. Additionally, many proteins lack corresponding antibodies, thereby limiting the applicability of quantitative proteome and transcriptome methods. To achieve a more comprehensive and qualitative understanding of cellular proteomes in conjunction with other omics approaches, the development of antibody-independent methods is imperative [98].

### 4.2 Mass cytometry-based protein measurements in multiomics

The extant procedure in single-cell multiomics technology employing mass spectrometry principally utilizes isotope-labeled antibodies for cell attachment, followed by inductively coupled plasma ionization to deconstruct the sample to its elemental constitution, liberate the isotope label, and subsequently channel it into the mass spectrometer. Through analysis, we can determine the abundance of proteins corresponding to different isotopes [98]. However, because of the limited number of lanthanide isotopes available, only a few proteins can currently be detected. PLAYR (proximity ligation assay for RNA) is a method based on mass cytometry that can quantitatively analyze mRNA and protein in the same cell simultaneously (Fig. 4). This technique bears a resemblance to protein detection methodologies grounded in PEA or PLA. However, it employs dual probes conjoined with isotopically labeled oligonucleotides targeting RNA, subsequently undergoing proximity ligation to engender circular DNA oligonucleotides amenable for subsequent rolling circle amplification. Given that proteins are tagged using metal isotope-labeled antibodies, both RNA and protein can concurrently be ascertained by mass cytometry. This approach enables the quantification of more than 40 different proteins and RNAs simultaneously.

One merit of methodologies predicated on mass spectrometry is their capability to execute proteomic spatial imaging [114,115]. Combining mass spectrometry imaging with spatial transcriptomics presents a compelling instrument for the concurrent analysis of transcripts and proteins within tissue sections. First, mass spectrometry tags are used to target proteins and RNA in cells, and then the labeled analytes are ionized according to specific trajectories for mass spectrometry analysis. Finally, the component information obtained in each area is repositioned, so that the spatial image of the target protein and RNA can be obtained. For example, spatial transcriptome and multiplexed ion beam imaging (MIBI) analysis of the same cutaneous squamous cell carcinoma tissue section can link the spatial distribution and abundance information of transcripts and proteins [116]. Recently, multiomics methodologies based on mass spectrometry imaging have been comprehensively reviewed [117]. Those with an interest in this topic are encouraged to consult the aforementioned review for further details.

### 4.3 Single-cell triomics integrating protein measurement

In all the above methods, at most, only two levels of the central dogma are involved. In recent years, several methods have spanned the central dogma to dissect the mechanism of cellular gene regulation and cell state from three modalities.

**Fig. 5:**
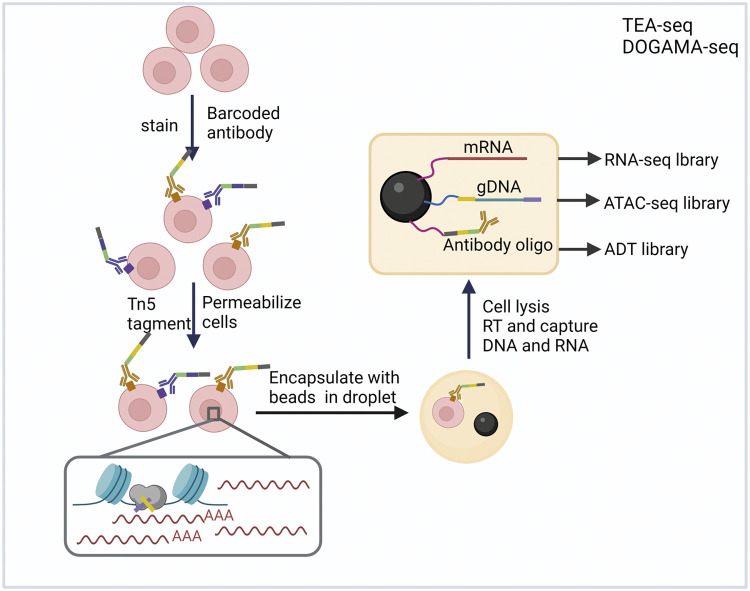
**TEA-seq and DOGMA-seq for the joint analyses of the epigenome, transcriptome, and proteome**.

The aforementioned scTrio-seq is limited in its inability to analyze protein levels and its low throughput capacity. In contrast, TEA-seq, when combined with the droplet-based multiomics platform can simultaneously analyze the transcriptome, chromatin accessibility, and cell surface proteins in thousands of single cells. In TEA-seq, the cell suspension is first immunostained with barcoded antibodies such as CITE-seq [35] to conjugate the surface proteome of interest. Cells were permeabilized followed by Tn5 tagmentation to the open region of chromatin. After completing the manual steps of these two procedures, single cells and beads with cell barcodes are encapsulated in GEMs (gel beads in emulsion) using an automated platform. The oligos and polyA tails present on the beads facilitate the capture of mRNA, antibody oligos, and open chromatin fragments and tags, all bearing the same cell barcode, resulting in the generation of cDNA, ADT (antibody-derived tag), and ATAC-seq libraries. (Fig. 5). DOGMA-seq also adopts a similar strategy, combining CITE-seq and scATAC-seq to jointly analyze chromatin accessibility alongside mitochondrial DNA mutation, transcriptome, and membrane proteins in single cells [104].

NEAT-seq is yet another methodology that spans the central dogma [118], enabling the concurrent profiling of nuclear protein quantification, open chromatin accessibility, and gene expression. In contrast to the previous two methods, NEAT-seq stains nuclear proteins isolated from single cells with a panel of master transcription factors (TFs) that drive T-cell subsets. NEAT-seq provides a new method to study the impact of transcription factor abundance on chromatin state and gene expression. To minimize background oligo-antibody staining in the nucleus, the charge of the antibody oligo is directly blocked using *E. coli* ssDNA binding protein (EcoSSB). Furthermore, the incorporation of hashtag oligo-linked antibodies (HTOs) allows for the parallel measurement of multiplexed proteome, epigenome, and transcriptome levels [119].

## 5 Data integration

The advent of single-cell multiomics technology has produced a substantial volume of experimental data from different modalities. While interpreting this vast dataset to derive meaningful insights poses a significant challenge, it is imperative to note that the discernment of biological insights from such analyses hinges upon the simultaneous development of fitting computational methodologies. To address this, a principal aim in refining single-cell multiomics data integration is to devise robust, sensitive computational strategies and statistical models. Specifically, these approaches are crafted to illuminate aspects such as cellular differentiation trajectories, genomic regulatory networks, intercellular interactions, cellular lineages, and other salient facets of cellular identity.

**Fig. 6:**
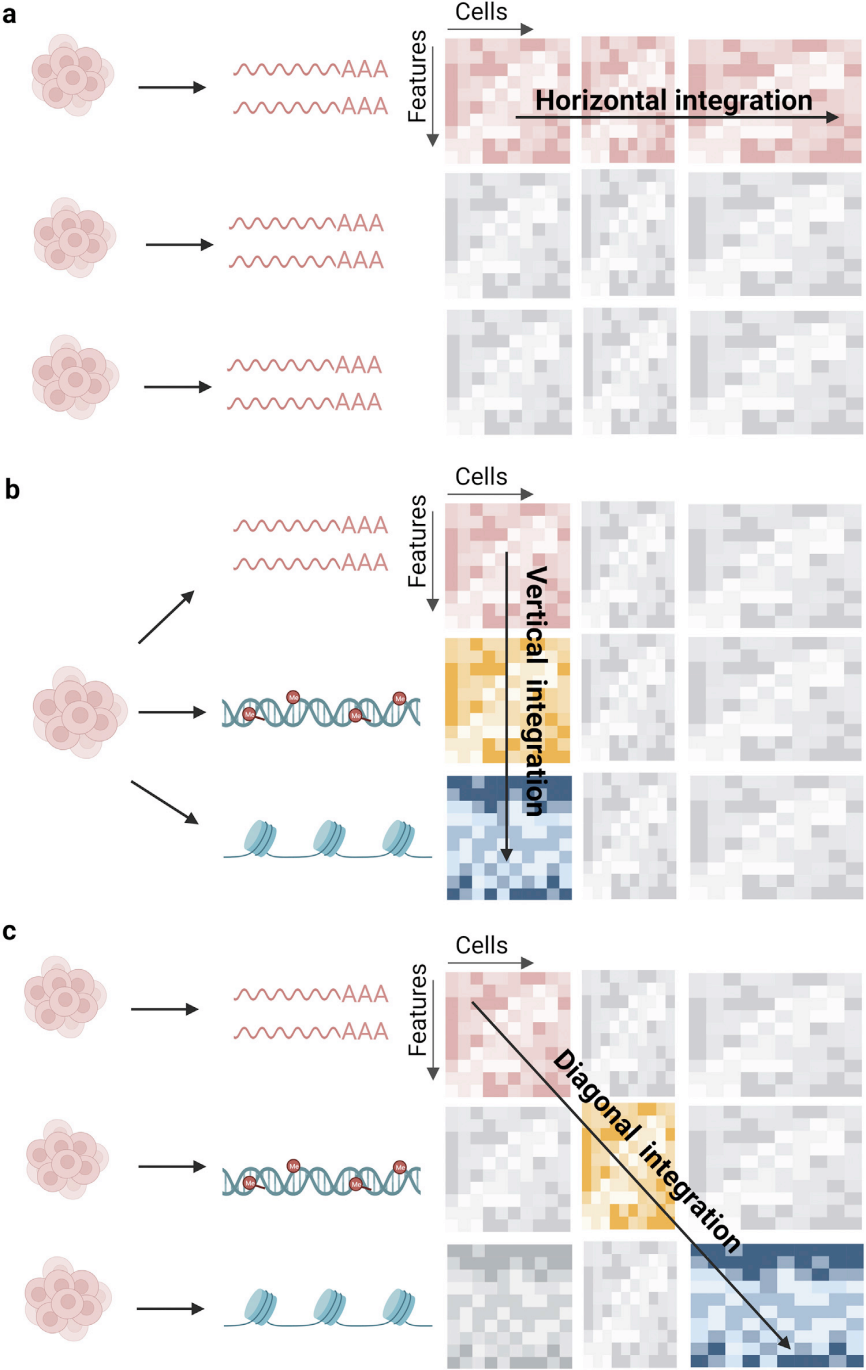
**Three strategies for single cell multiomics data processing.** (a) Horizontal integration, which utilizes cells as anchors. (b) Vertical integration, which utilizes features as anchors. (c) Diagonal integration, in which there is no anchor.

In alignment with the aforementioned objectives, a suite of data integration methodologies for single-cell multiomics has been established. Based on distinctive anchors that bridge various data modalities, Argelaguet and colleagues have classified these methods into three distinct categories [120]. The first category is horizontal integration. This approach employs genomic features as anchors to examine identical data modalities garnered from disparate cell populations or via various technological methodologies. The second category is vertical integration which utilizes an individual cell as the anchor. This method focuses on analyzing diverse data modalities derived from the same single cell. The third category is diagonal integration. (Fig. 6). This methodology operates without a specific anchor, signifying that the data originate from varied cells and encompass diverse modalities. Within these classifications, both horizontal and diagonal integrations are categorized as unmatched assays, whereas vertical integration is a matched assay. The integration of nonmatched data poses greater challenges than matched data, as it necessitates the elimination of technical discrepancies across diverse datasets while retaining data of biological significance within or across experiments. A suite of methods has been developed for the integration of nonmatched data, such as Seurat v3 [121], LIGER [122], UnionCom [123], MultiVI [124], GLUE [125], and Seurat v5 [126]. These methodologies ameliorate the aforementioned issues to varying degrees. However, a majority of these techniques also encounter obstacles. Firstly, there's a risk of overcorrection when the correction vectors are inaccurately estimated, and a common consequence is the loss of valuable biological variation information within the data. Second, in most of these methods, embedding in a latent space is imperative, yet this can lead to data distortion due to batch alignment, subsequently affecting downstream analytical outcomes. Compared to the integration of nonmatching data, the integration of matching data is relatively more straightforward, given that the multimodal data originates from the same cell, obviating the need to address batch variability. Recently developed methodologies in this category include citeFUSE [127], totalVI [128], DeepMAPS [129], SCENIC+ [130], Seurat v4 [131], and MultiVelo [132]. These methodologies also present certain challenges. First, molecular readouts gathered using diverse techniques exhibit distinct statistical characteristics. For instance, in scTrio-seq, differing data can be obtained from the genome, transcriptome, and methylome of the same cell. However, processing these data within a unified statistical framework is a complex endeavor. Second, some methods yield features with pronounced quantitative disparities. For example, the feature counts from the antibody libraries and transcriptome libraries in CITE-seq are not of the same order of magnitude. This results in embeddings within the latent space that may produce mappings disproportional to the modalities' quantities. Hence, there is a pressing need for the development of more robust and encompassing methodologies for the analysis of both matched and unmatched data in forthcoming research, with a particular need for the analysis of unmatched data.

## 6 Conclusion and perspectives

In a remarkably short span of time, significant progress has been made in the field of multimodal omics, driven by advancements in single-cell platforms and cell sorting methods. These advancements have enabled the profiling of hundreds, and even millions, of individual cells, offering unprecedented throughput, cost reduction, and the ability to investigate multiple modalities within the same single cell. This global perspective facilitates the comprehensive exploration of cellular types and states [133]. Expanding single-cell multiomics approaches to tissue samples holds great promise for cancer biology research, yet it also underscores the pressing need for higher-throughput sequencing methods.

While commercial single-cell high-throughput sequencing platforms and combinatorial indexing strategies have been widely adopted, the current analysis at each molecular level is not without limitations. Currently, most single-cell multiomics approaches are still in the early stages of development and have yet to achieve high-throughput capture of all molecular layers with high selectivity and sensitivity. Therefore, the key challenge is the balance between data sparsity and throughput. Subsequently, the signal-to-noise ratio of single-cell measurements remains suboptimal. It is currently difficult to discern cell-to-cell variability from technical noise, and single-cell high-throughput sequencing coverage remains low, which can lead to the loss of crucial signals related to mutations, modifications, and gene expression. While long-read sequencing methodologies can yield more comprehensive genomic information, they remain unsuitable for clinical sample analysis. The recently developed scNanoCOOL is a method based on long-read sequencing that might provide deeper insights into the functions of CpG islands and promoters in gene expression regulation [134]. Existing technologies predominantly focus on detecting various factors associated with the genome and epigenome, such as gene mutations, chromosome copy numbers, DNA methylation, nucleosome positioning, and chromosome accessibility. Although the relationship between epigenetic regulatory factors and transcription has improved our understanding of cell heterogeneity at the transcriptomic level, more modalities need to be integrated, including the same molecular layer and different modalities. Among multiomics techniques, the transcriptome has been the most extensively studied, serving as a vital link within the central dogma. However, current approaches primarily focus on readily available polyA RNAs, warranting further exploration of other types of RNAs that play crucial roles in cellular biological processes. The recent development of a droplet-based single-cell total RNA sequencing platform may be an ideal solution [135]. Additionally, while single-cell multimodal omics research has made progress in the integrated analysis of DNA 5mC epigenetic modifications and gene expression, investigations into other modifications, particularly RNA modifications, are still in their early stages. Recently, a method for the combined analysis of m^6^A methylation and the transcriptome was first reported, named sn-m6A-CT (single-nucleus m^6^A-cleavage under targets and tagmentation). This method was confirmed to be sufficient to determine cell identity and allows for the generation of cell-type-specific m^6^A methylome landscapes from heterogeneous populations [136]. This method has paved a new path for epitranscriptomics, and with technological advancements, we believe that more research findings in the future will focus on the integrated analysis of RNA modifications and other omics studies. Notably, while the significance of proteins as primary executors of biological functions within cells is undeniable, most mature protein multiomics methods presently focus on membrane proteins, with investigations into nuclear proteins only emerging in the last two years. These methods rely on the specific recognition of the protein of interest by barcoded antibodies. However, not every antibody exhibits optimal specificity, and not all can be efficaciously conjugated with oligonucleotides. This constrains the diversity of proteins amenable to analysis using this technique. Another significant limitation associated with the multiplexing analysis of proteins is that cross-reactivity among distinct antibodies can engender background noise. Mass spectrometry at the single-cell level presents an optimal selection for protein analysis, catering to both qualitative and quantitative evaluations. However, contemporary methodologies in single-cell mass spectrometry necessitate refinements to ensure compatibility with other high-throughput omics techniques. The decline in sequencing expenditures has catalyzed the production of single-cell multiomics datasets. However, the expansive analysis and amalgamation of these datasets necessitate the formulation of robot computational and statistical methodologies, with machine learning-based models presenting as quintessential instruments. Furthermore, it is imperative to establish standardized protocols for data analysis and to benchmark nascent computational tools, ensuring a rigorous evaluation of their efficacy.

It is evident that this field is poised to rapidly advance toward spatial multiomics, which can provide the spatial coordinates of cells and trace the location of cells within tissues without the need for single-cell dissociation or suspension, as in previous methods. A range of imaging-based or sequencing-based spatial multiomics methods have been developed [137], [138], [139], [140], [141], [142]. Based on these methods, we can foresee the future direction of this field. 1) The combination of high-throughput sequencing and mass spectrometry-based imaging methods allows for the study of more omics-level interactions at the tissue level simultaneously, including but not limited to metabolome. 2) Methods compatible with commercially available platforms will be developed. 3) Robust and accurate data analysis methods based on machine learning or artificial intelligence will be developed. These advances will revolutionize our understanding of biological processes and provide more new targets and biomarkers for drug development and precision medicine.

## Declaration of competing interest

The authors declare that they have no conflicts of interest in this work.
